# Isolated Prothrombin Deficiency: A Case Report of a Rare Coagulation Disorder and Review of Literature

**DOI:** 10.7759/cureus.55940

**Published:** 2024-03-11

**Authors:** Pranathi Bollineni, Febe Renjitha Suman, Dhaarani Jayaraman, Nivedha Subramani, Sudeep Gaddam

**Affiliations:** 1 Pediatrics, Sri Ramachandra Institute of Higher Education and Research, Chennai, IND; 2 Pathology and Laboratory Medicine, Sri Ramachandra Institute of Higher Education and Research, Chennai, IND; 3 Pediatric Hematology, Oncology, and Bone Marrow Transplant, Sri Ramachandra Institute of Higher Education and Research, Chennai, IND; 4 Pediatric Medicine, Sri Ramachandra Institute of Higher Education and Research, Chennai, IND; 5 Pediatric Oncology, Sri Ramachandra Institute of Higher Education and Research, Chennai, IND

**Keywords:** activated partial thromboplastin time, balanitis xerotica, factor ii deficiency, coagulation disorder, prothrombin deficiency

## Abstract

Congenital prothrombin deficiency is a rare hemorrhagic disorder, frequent in areas with high degrees of consanguinity as it is autosomal recessive in nature. Clinical manifestations are highly variable, ranging from mild episodes of bleeding to severe hemorrhages. Here, we report a child with isolated prothrombin deficiency who presented with a history of pain and soreness in the prepuce associated with bleeding. Laboratory evaluation showed an altered coagulation profile with a prothrombin activity level of 29.8%, indicative of factor-II deficiency. This case highlights the importance of coagulation screening in all patients before even minor invasive procedures and the role of a detailed coagulation profile in confirming a diagnosis in the case of abnormal screening tests.

## Introduction

Congenital prothrombin deficiency, or factor-II deficiency, is an extremely rare disorder of coagulation or a rare bleeding disorder. This has an autosomal recessive pattern of inheritance and hence is more common in areas with high degrees of consanguinity, which usually presents with a wide range of clinical manifestations, from asymptomatic mild bleeding episodes to life-threatening spontaneous or post-traumatic hemorrhages. It has been described that a complete prothrombin deficiency is incompatible with life [1]. Apart from bleeding complications in type-1, type-3 prothrombin deficiency can cause thrombotic complications due to defective binding to antithrombin [2]. Here, we report a case of isolated prothrombin deficiency affecting factor-II in the coagulation pathway.

## Case presentation

An eight-year-old boy, born to a second-degree consanguineously married couple, presented with a history of pain and soreness of preputial skin. The patient was diagnosed with balanitis xerotica obliterans. Circumcision was planned by pediatric surgeons, and he was admitted for surgery. He had bleeding with prolonged oozing from the preputial skin, requiring pressure for 5-10 minutes to stop bleeding, which was attributed to the local inflammation and infection. There was no other significant past medical history, including prolonged bleeding after any trauma or tooth fall. No history of hematoma after vaccinations or significant bleeding history in the family was reported. No history of umbilical cord bleeding or any other bleeding was reported in the early neonatal period.

The baseline screening coagulation profile before circumcision showed a prolonged activated partial thromboplastin time (APTT) of 44.4 seconds and a prolonged clot-based prothrombin time (PT) of 15.2 seconds. A complete hemogram was normal. As there was no significant bleeding history, values were repeated after three days of vitamin K supplementation. Repeat analysis still showed high APTT and PT values of 44.5 seconds and 15.2 seconds, respectively. Hence, mixing studies were done; upon mixing with normal plasma, a PT value of 12 seconds and an APTT value of 28.2 seconds were generated, which means mixing corrected the prolongation, suggesting factor deficiency.

A detailed coagulation profile further revealed a prothrombin activity level of 29.8%, indicative of mild factor-II deficiency. Other factors, including Von-Willebrand activity, were within the normal range. Parents were counseled about the nature of the disease and the risk of bleeds. The patient then underwent surgery under the cover of tranexamic acid and strict hemostatic measures. No other intervention was required as there were no significant bleeding complications in the postoperative period. The family screening was suggested, but couldn’t be done due to logistical reasons.

## Discussion

Prothrombin deficiency, one of the rarest coagulation disorders, was first discovered in 1947 by Dr. A Quick [3]. Factor-II (prothrombin) is synthesized in the liver and circulates in the bloodstream as an inactive precursor to thrombin, an enzyme that has both positive and negative feedback mechanisms on different steps of the coagulation cascade. It is responsible for converting fibrinogen to its active form, fibrin, to stabilize a blood clot [4]. It is a vitamin K-dependent protein encoded by a gene on chromosome 11p11.q1212 [5]. The factor has a half-life of around three days [6].

Patients with prothrombin activity of less than 5% have severe symptoms presenting earlier in life; patients with 5-10% have moderate symptoms; and patients with greater than 10% have mild symptoms. The tendency to bleed is generally inversely proportional to the level of factor-II activity, as in hemophilia [3]. Mild coagulation defects are often clinically silent and are evident after significant trauma or while screening for invasive procedures or surgery with a baseline coagulation screening profile. Simultaneous prolongation of PT and APTT, along with decreased factor-II activity, is useful for confirmation [7].

There are no individuals with completely undetectable prothrombin levels, as it is not compatible with life. Activated partial thromboplastin time and prothrombin time prolongation should lead to the differentials, including factor-II deficiency and factor-II assay, being reduced while other vitamin K-dependent factors are normal [8]. In our patient, the prothrombin activity level was 29.6%, indicative of mild isolated factor-II deficiency.

This entity has been divided into hypoprothrombinemia, or “true deficiency” (type 1), dysprothrombinemia (type 2), and hypoprothrombinemia without bleeding but with thrombosis (type 3). In type 1 prothrombin deficiency, both prothrombin levels and prothrombin activity are reduced; in type 2 deficiency, prothrombin activity is reduced but prothrombin levels are in the borderline range; whereas, in type 3, prothrombin deficiency can have paradoxical thrombotic complications due to defective binding of antithrombin. Although most patients with hypoprothrombinemia have been reported to exhibit a bleeding phenotype, mutations have led to thrombotic episodes in some cases; thus, classifying the condition as a hemorrhagic-thrombotic disorder depending on the type of mutation present has gained importance [9].

There has been little progress in the development of factor-II concentrates since 1947 due to the rarity of factor-II-deficient cases with unclear guidelines on both prophylaxis and treatment [10]. In children with life-threatening complications due to severe prothrombin deficiency, prophylactic treatment is suggested to prevent episodes of bleeding. Treatment to manage the condition includes the administration of prothrombin complex concentrates or fresh frozen plasma, which may be required as per the short three-day half-life of prothrombin [11]. In mild to moderate episodes of bleeding, anti-fibrinolytic therapies such as tranexamic acid may be administered orally or intravenously for treatment [6].

## Conclusions

This case highlights the importance of coagulation screening in all patients before invasive procedures and the need for a detailed coagulation profile to confirm the diagnosis in cases of abnormal screening tests. Mild coagulation defects may typically go unnoticed with no clinical evidence and may only be detected with detailed screening tests before invasive procedures or surgery.
